# Clinical Outcomes of Anterior Cruciate Ligament Reconstruction Using Quadriceps Tendon Autograft Versus Hamstring Tendons Autograft: A Retrospective Analysis

**DOI:** 10.7759/cureus.18760

**Published:** 2021-10-13

**Authors:** Mohd A Aslam, Sachin Avasthi, Pankaj Aggarwal, Satyam Singh, Vineet Kumar, Swagat Mahapatra

**Affiliations:** 1 Department of Orthopaedic Surgery, Dr. Ram Manohar Lohia Institute of Medical Sciences, Lucknow, IND

**Keywords:** modified cincinnati knee score, tegner-lysholm knee scoring system, hamstring tendon, quadriceps tendon, acl reconstruction

## Abstract

Aim: The purpose of this retrospective study was to examine the clinical outcomes of anatomic single-bundle anterior cruciate ligament (ACL) reconstruction using a free quadriceps (QUADRI) tendon or a quadrupled hamstring (HAM) autograft.

Material and method: The retrospective analysis (Level III, Therapeutic Study) included consecutive patients who underwent ACL reconstruction between April 2017 and April 2020 using either a free quadriceps tendon autograft or a hamstring tendon autograft. All patients underwent ACL reconstruction to treat isolated ACL injuries. The Tegner-Lysholm knee scoring system and the modified Cincinnati knee score were used for evaluation before surgery, and at six weeks, six months, and one-year follow-up time.

Results: In the present study, 35 people underwent quadriceps (QUADRI) grafts and 35 underwent hamstring (HAM) grafts. The demographic data for the groups were extremely comparable. The mean follow-up length for the HAM group was 11.96±0.28 months, while the QUADRI group had a mean follow-up period of 11.25±0.43 months. No significant variations in the Cincinnati score were observed between the two groups during any of the treatment's follow-up periods. Similarly, the Tegner Lysholm Score revealed no statistically significant differences between clinical outcomes in the HAM and QUADRI groups at all follow-up visits, except for the sixth week.

Conclusion: Clinical outcomes are comparable in terms of stability and subjective assessments following ACL reconstruction using a free quadriceps or hamstring tendon autograft.

## Introduction

The hamstring tendon (HAM) autograft is used most often for autograft anterior cruciate ligament (ACL) reconstruction [1], followed by bone-patellar tendon-bone (BTB) autograft [2]. Both graft approaches have advantages and disadvantages, as there is no conclusive proof that one is preferable over the other [3-4]. However, in terms of graft-related morbidity, HAM grafts are recognised to cause less donor site morbidity than BTB grafts [5-6].

Recently, interest in using quadriceps tendon (QUADRI) as an autologous graft for ACL reconstruction has been established [7]. This increased interest could be attributed to the advancement of graft harvesting procedures, which have resulted in the introduction of less invasive treatments using smaller incisions [8]. However, despite the fact that the QUADRI as graft has been used for ACL reconstruction for a lengthy period of time and with positive results, it is still regarded as a secondary alternative for primary ACL reconstruction [9]. Several studies indicate that donor site morbidity is significantly lower following QUADRI-ACL surgery than BTB-ACL reconstruction [7]. Additionally, donor site morbidity was found to be significantly lower for the free quadriceps transplant, without a patellar bone block, than for the HAM graft harvest [10].

Additionally, significant conceptual advantages of QUADRI autografts include their consistent size, their adaptability, and the capacity to harvest grafts in a variety of widths, thicknesses, and lengths [8,11]. Additionally, graft maturity was found to be superior at six months following ACL reconstruction using QUADRI versus HAM autograft. However, this study used a bone-QUADRI graft [11]. Still, there is a dearth of data comparing the clinical results of patients who underwent ACL reconstruction using a free QUADRI or HAM autograft. The goal of this study was to compare the clinical results of patients undergoing anatomic single-bundle (SB) ACL reconstruction with a free QUADRI autograft against a quadrupled HAM autograft over a one-year follow-up.

## Materials and methods

Study design

This retrospective study (Level III, Therapeutic Study) was carried out between April 2017 and April 2020 in our institution's orthopaedic outpatient clinic, where 96 patients underwent ACL replacement using quadrupled hamstring (HAM) or quadriceps tendon (QUADRI) autogenous grafts. The study enrolled 70 individuals who met the inclusion criteria and were followed on a regular basis. This study was granted approval on February 19, 2021, by the Institutional Ethical Committee, Dr. Ram Manohar Lohia Institute of Medical Sciences, Lucknow, with approval number RC. 217/21/RMLIMS/2021.

Inclusion exclusion criteria

All patients of either gender or any age undergoing ACL reconstruction utilising quadruple hamstring or quadriceps tendon patellar bone grafts were included in the study. Patients with concomitant cartilage lesions, meniscal lesions requiring meniscectomy or repair, multi-ligamentous lesions, and patients who had previously undergone knee surgery on the afflicted or contralateral knee (absolute distance) were excluded.

Study groups

The study included 70 patients segregated randomly (computer-generated) into two groups. In which 35 patients underwent ACL reconstruction utilising quadruple hamstring grafts and 35 received quadriceps tendon patellar bone grafts.

Procedure

Along with clinicodemographic data collection, all enrolled patients underwent arthroscopic ACL reconstruction. All reconstructions were performed using a single bundle ligament. As a graft, an ipsilateral hamstring (HAM) or quadriceps (QUADRI) tendon was taken. A 2.5 to 3 cm transverse incision was made above the superior border of the patella to harvest the quadriceps tendon. After incising the suprapatellar bursa, the quadriceps tendon is revealed. At the middle of the superior border of the patella, a specific double knife with a width of 8 to 12 mm is inserted and pushed up to 8 cm proximal to the starting site.

After determining the thickness, a specific tendon separator was used to elevate the graft that was subsequently separated from its proximal attachment using a special tendon cutter, and then the graft was retrieved. The graft was subperiosteally separated from the patella at its distal connection. The periosteal end of the graft was folded in half and stitched with a strong fibre wire to round it off and facilitate graft transit. Sutures were then threaded through a flip button device and secured subsequently. In both groups of patients, the graft was secured on the femoral side using a flip button device and on the tibial side with a bioscrew.

Examinations and follow-ups

Patients took a single-leg hop distance test by jumping as far as possible on one leg while maintaining balance and landing firmly. The distance between the starting line (meter) and the heel of the landing leg was calculated. At 0-2 weeks, mobilization of patients with the help of axillary crutches was done. Static quadriceps and hamstring exercises, closed chain exercises were followed. At 2-6 weeks, prone hangs and passive full flexion of the knee, static hamstring and quadriceps exercised, half squats were instructed. Furthermore, at six weeks to three months, slow forms running, quadriceps and hamstring strengthening were done. Lastly, the patients gradually returned to sports activities after 3-6 months.

All patients were evaluated using the Tegner-Lysholm knee grading system and the modified Cincinnati knee score prior to surgery and during the sixth week, the sixth month, and one-year follow-up. The Lachman score [12] and single hop test were performed at the end of one year. A single person carried out the Lachman test every time, and an arthrometer was not used. Muscle strength testing was not done.

Statistical analysis

Statistical analysis was performed using SPSS software for Windows, version 15.0 (SPSS Inc., Chicago, USA). Dichotomous variables were measured in proportions and continuous variables were measured as mean and standard deviation. Chi-squared test, as applicable, was used to measure the association between proportions. The difference in continuous variables was measured using paired/unpaired t-test, as applicable. A p-value of less than 0.05 was taken statistically significant. 

## Results

**Figure 1:**
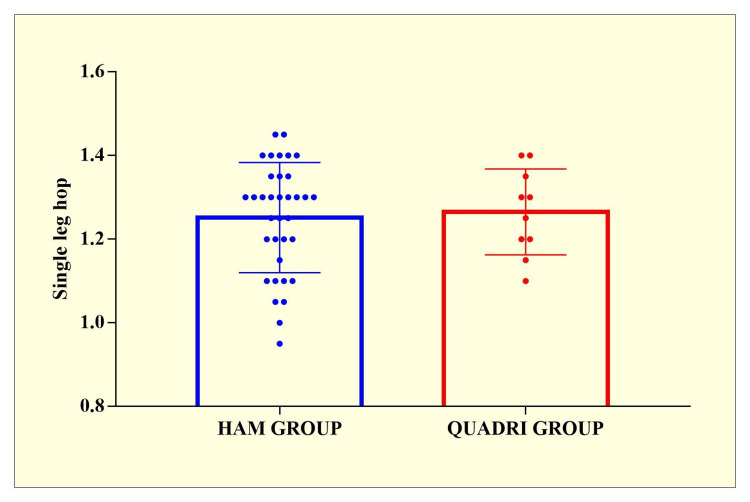
Single leg hop analysis between HAM and QUADRI groups. HAM group - hamstring tendon autograft used for anterior cruciate ligament (ACL) reconstruction; QUADRI group - quadriceps tendon autograft used for ACL reconstruction

**Figure 2:**
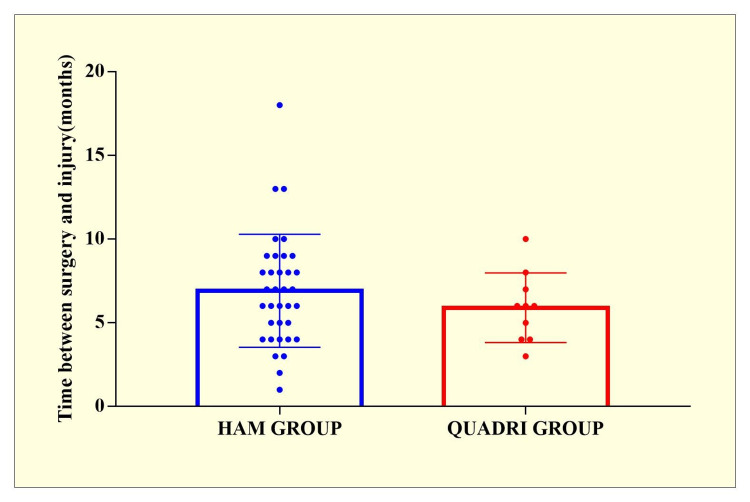
Time between surgery and injury in HAM and QUADRI groups. HAM group - hamstring tendon autograft used for anterior cruciate ligament (ACL) reconstruction; QUADRI group - quadriceps tendon autograft used for ACL reconstruction

A total of 70 respondents were seen in the hospital for a follow-up after one year. Total 35 individuals had quadriceps (QUADRI ) grafts, and 35 had hamstring (HAM) grafts. The demographic data for the groups were extremely comparable, as seen in Table 1 and Figures 1-2. The mean follow-up length for the HAM group was 11.96±0.28 months, while the QUADRI group had a mean follow-up period of 11.25±0.43 months. There were no statistically significant differences in Single leg hop and Lachmann grade scores between the two groups. There were no readmissions or re-operations for problems in either group.

**Table 1:** Demographical representation of HAM and QUADRI groups. HAM group - hamstring tendon autograft used for anterior cruciate ligament (ACL) reconstruction; QUADRI group - quadriceps tendon autograft used for ACL reconstruction; t= Student's t-test (Unpaired t-test); X= Chi-Square Test; ^¥^p= p-value from Chi-Square test

Parameters		HAM Group (Mean± SD) [n=35]	QUADRI Group (Mean± SD) [n=35]	Statistical test and p-values
Age		25.01 ± 4.10	24.6 ± 3.3	t=0 p>0.9999
Gender	Male	28 (80%)	19 (90%)	X=2.241 ^¥^p=0.1344
	Female	7 (20%)	11 (10%)	
Time between surgery and injury (months)		6.91 ± 3.37	5.9 ± 2.07	t=0.8991 p=0.3736
Single leg hop		1.251 ± 0.13	1.265 ± 0.10	t=0.3004 p=0.7653
Lachmann grade		1±0	1±0	--

While analysing the Cincinnati score in both groups, no significant differences between both groups were observed during follow-up as shown in Table 2 and Figure 2.

**Table 2:** Cincinnati score comparison between HAM and QUADRI groups. HAM group - hamstring tendon autograft used for anterior cruciate ligament (ACL) reconstruction; QUADRI group - quadriceps tendon autograft used for ACL reconstruction; Pre-Op - Pre-operation; t= Student's t-test

Follow-up time	HAM Group (Mean± SD) [n=35]	QUADRI Group (Mean± SD) [n=35]	Statistical test and p-value
Pre-Op	33.65±4.9 (0.84)	32.82 ±4.8 (1.54)	t=0.4744 p=0.6376
Six weeks	64.9±5.9 (0.99)	62.8±5.43 (1.71)	t=1.009 p=0.3187
Six months	71.22±5.05 (0.85)	69.8±3.44 (1.09)	t=0.8323 p=0.4099
One Year	75.28±4.2 (0.731)	75.11±2.35 (0.74)	t=0.122 p=0.9035

**Figure 3:**
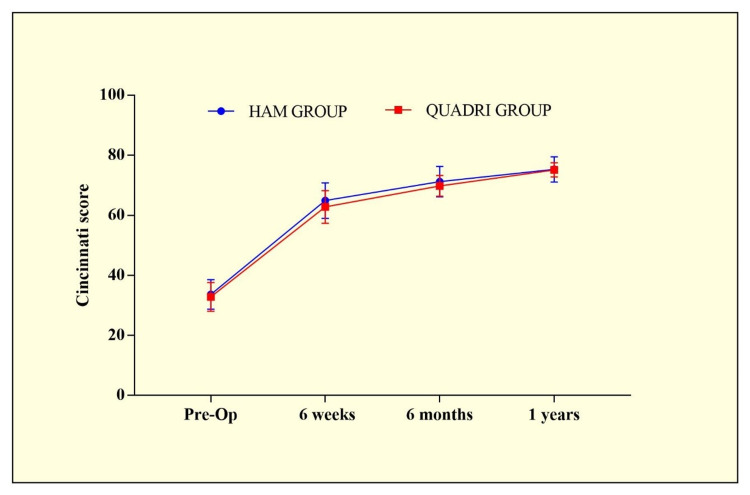
Cincinnati score comparison between HAM and QUADRI groups. HAM group - hamstring tendon autograft used for anterior cruciate ligament (ACL) reconstruction; QUADRI group - quadriceps tendon autograft used for ACL reconstruction

**Figure 4:**
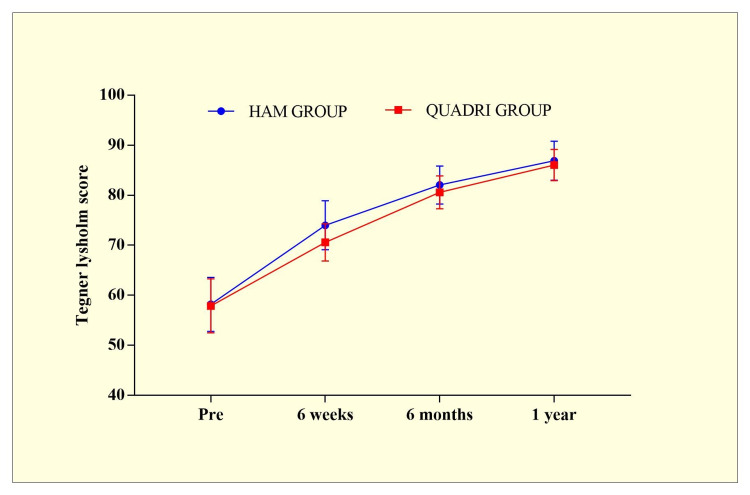
Tegner Lysholm score between HAM and QUADRI groups. HAM group - hamstring tendon autograft used for anterior cruciate ligament (ACL) reconstruction; QUADRI group - quadriceps tendon autograft used for ACL reconstruction

Similarly, Tegner Lysholm score also showed insignificant differences in all follow-up, except at the sixth week, in both HAM and QUADRI groups (Table 3 and Figure 4). 

**Table 3:** Tegner Lysholm Score between HAM and QUADRI groups. HAM group - hamstring tendon autograft used for anterior cruciate ligament (ACL) reconstruction; QUADRI group - quadriceps tendon autograft used for ACL reconstruction; Pre-Op - Pre-operation; t= Student's t-test

Follow-up time	HAM Group (Mean± SD) [n=35]	QUADRI Group (Mean± SD) [n=35]	Statistical test and p-value
Pre-Op	58.2±5.4 (0.916)	57.9±5.4 (1.73)	t=0.1549 p=0.8776
Six weeks	74±4.9 (0.82)	70.6±3.74 (1.1)	t=2.026 p=0.0490*
Six months	82.05±3.8 (0.64)	80.6±3.27 (1.08)	t=1.094 p=0.2799
One Year	86.9±3.89 (0.657)	86.04 ±3.1 (0.916)	t=0.6415 p=0.5246

## Discussion

While the quadriceps-patellar bone (QUADRI-PB) graft is one of the autogenous graft choices that has been examined and debated scientifically, it is less popular than the HAM graft. It is often preferable in revision surgery or when many ligaments are injured. From a biomechanical standpoint, a central quadriceps tendon autograft, with or without a patellar bone plug, was demonstrated to be a viable choice for ACL reconstruction surgery [4].

In the present study, a total of 35 individuals had quadriceps (QUADRI) grafts, and 35 had hamstring (HAM) grafts. Significant differences were observed between the HAM and QUADRI groups during the clinical examination at the sixth-week follow-up. However, on further follow-ups, no significant differences were observed. When the Cincinnati score was compared between the two groups, no significant differences were seen at either of the treatment's follow-up periods. Similarly, the Tegner Lysholm score demonstrated no statistically significant differences among clinical observations in all follow-up visits, except for the sixth week, in both the HAM and QUADRI groups.

To our knowledge, just one study, done in Romania, by Todor et al. (2019), compared the clinical outcomes of these two distinct autografts [13]. Todor et al. study's primary findings indicated that comparable results in terms of stability and patient-reported outcomes could be reached using either a HAM or a free QUADRI autograft [13]. They revealed no statistically significant differences between groups in terms of instrumented laxity tests, Lysholm scores, and modified Cincinnati scores. Muscle recovery is another critical component of graft selection. Todor et al. found a statistically significant difference between the QUADRI and HAM groups in terms of thigh muscle atrophy. However, muscle recovery was not assessed in terms of strength, and pre-operative thigh measurements were not documented [13]. The present study observations are comparable to the Todor et al. [13] study.

Iriuchishima et al. demonstrated comparable muscle recovery following ACL reconstruction with QUADRI against previously reported data using HAM autografts [14]. Fischer et al. showed a statistically significant decrease in knee extensor strength and an increase in flexor muscle strength in the QUADRI group compared to the HAM group following ACL reconstruction with quadriceps grafts versus hamstrings graft [15]. Additionally, patients receiving QUADRI grafts had a greater hamstring/quadriceps (H/Q) ratio within the first few months following surgery. Similarly, Todor et al. [13] found no statistically significant difference between reconstruction using a free quadriceps or hamstring tendon autograft reconstruction. The present study found similar observations.

This study clinically validates the use of a free QUADRI graft fixed on the femur with an extra-cortical button attached to the graft with high strength sutures, a technique previously described in the literature [16]. A recent study by Runer et al. showed similar results, with no difference between QUADRI and HAM autografts in patients with ACL reconstruction at two-year follow-up [17]. However, the authors used bone-QUADRI grafts. Another study by Cavaignac et al. showed equal or better functional outcomes with bone quadriceps graft compared to hamstrings graft ACL reconstruction after more than three years [18]. Using a free QUADRI graft can minimize donor site morbidity without compromising the results. Overall, donor site morbidity has been found to be minimal with the quadriceps graft, both with a normal or minimally invasive harvesting technique [7,10,14]. Still, QUADRI is the least used graft for primary ACL reconstruction, with about 10% of the reconstructions being performed with a quadriceps graft [18]. It is expected that the use of this graft will be increasing in the future [19] as data shows good anatomical and biomechanical characteristics to the QUADRI graft [17,19]. Also, studies have shown good clinical results with QUADRI graft compared to patellar tendon graft still considered the gold standard by some authors. Lund et al. found comparable results in a prospective randomized trial comparing QUADRI with a patellar tendon [20]. However, knee walking pain was significantly less for QUADRI than with BTB. Similar results were reported by others [16]. In a systematic review by Slone et al., which included 14 studies of which six compared QUADRI grafts versus BTB grafts, there were similar results regarding laxity, functional outcomes, overall patient satisfaction, range of motion (ROM), and complications between QUADRI and other graft options [7]. A recent article by Belk et al. reported less knee laxity in patients with QUADRI-ACL reconstruction compared to HAM patients but with no difference in failure rates between groups [21]. Other advantages may be attributed to the QUADRI graft. A study based on magnetic resonance imaging by Ma et al. showed that graft maturity was better at six months following ACL reconstruction with QUADRI compared to the HAM autograft [11].

The strengths to be noted with the study are the homogeneity of the groups in terms of demographics and the fact that pure ACL reconstructions were selected, without associated meniscal or cartilage procedures that could have influenced the outcomes. Further, the same surgical technique was used throughout the study and by the same operating surgeon. However, the study has several limitations to be considered. First, it is a retrospective study with the documented clinical examination at the last follow-up. Also, the follow-up duration was short (only one year). The person who collected the data was also not blinded to the graft used. Furthermore, the graft choice was not randomized, and the decision was made by the operating surgeon after discussing it with the patients. The authors recommend further multicentric prospective studies with large samples to increase the reliability and generalizability of the study.

## Conclusions

No statistically significant difference in outcomes was detected when the reconstruction was performed using a free quadriceps (QUADRI) or hamstring (HAM) tendon autograft. This means that compared clinical outcomes in terms of stability and subjective parameters can be obtained following ACL reconstruction using a free quadriceps or a four-strand hamstring tendon autograft.
